# Response to comment on 'A conserved strategy for inducing appendage regeneration in moon jellyfish, *Drosophila*, and mice'

**DOI:** 10.7554/eLife.85370

**Published:** 2023-06-22

**Authors:** Yutian Li, Anish A Sarma, Iris T Lee, Fayth Hui Tan, Michael J Abrams, Zevin J Condiotte, Martin Heithe, Misha Raffiee, John O Dabiri, David A Gold, Lea Goentoro

**Affiliations:** 1 Division of Biology and Biological Engineering, California Institute of Technology Pasadena United States; 2 Graduate Aerospace Laboratories and Mechanical Engineering, California Institute of Technology Pasadena United States; CNRS - Université Paris Cité France; University of Michigan United States

**Keywords:** moon jellyfish, regeneration, insulin, leucine, appendage, *D. melanogaster*, Mouse

## Abstract

Previously we reported evidence that a regenerative response in the appendages of moon jellyfish, fruit flies, and mice can be promoted by nutrient modulation (Abrams et al., 2021). Sustar and Tuthill subsequently reported that they had not been able to reproduce the induced regenerative response in flies (Sustar and Tuthill, 2023). Here we discuss that differences in the amputation method, treatment concentrations, age of the animals, and stress management explain why they did not observe a regenerative response in flies. Typically, 30–50% of treated flies showed response in our assay.

## Introduction

An increasing body of evidence suggests that animals can be coaxed to regenerate better. In adult frogs, studies dating back to early 1900s (Carlson, 2007) demonstrate that limb regeneration can be induced by salt treatment, electrical stimulation, and in more recent studies, progenitor cell implantation (Lin et al., 2013) and drug cocktails (Murugan et al., 2022). In fish, cardiomyocyte proliferation in the medaka can be induced by acute inflammation (Lai et al., 2017). Even in adult mice, a regenerative response can be promoted in multiple organs, including the heart (Sadek and Olson, 2020), retinal axon (Bei et al., 2016), and limbs (Yu et al., 2012). Adding to this body of work, we presented evidence that adult *Drosophila* limb can be promoted to show regrowth by administering insulin and amino acids (Abrams et al., 2021). Although none of the induced regeneration is complete, these studies demonstrate the biological phenomenon that, in multiple species, adult animals retain a capacity to mount a regenerative response greater than they normally show.

Sustar and Tuthill report that they were unable to reproduce our observations in the fly limb (Sustar and Tuthill, 2023). Reading their method, we find multiple, significant differences in the way Sustar and Tuthill performed our protocol. The differences are parameters known to alter outcomes in regeneration experiments, and consequently are parameters that we have carefully controlled over the past five years of developing and performing this protocol. Here we explain why these factors—injury method, animal age, treatment, and stress management—can alter the outcome of regeneration experiments.

## Results

Comparing our protocol to that of Sustar and Tuthill, we identify critical differences in six areas: amputation method; age of the flies; treatment concentrations; anesthesia protocol; housing density; regeneration assessment.

### Amputation method

Amputation method is a key parameter that can profoundly alter the outcomes of regeneration processes. A significant portion of the regeneration literature is dedicated to defining and comparing “injury models” (e.g., for muscle injury, see Hardy et al., 2016; Sicherer et al., 2020; for heart injury, see Luther et al., 2013; for brain injury, see Xiong et al., 2013). For instance, in studies of muscle repair in mice, injury models vary from surgically resecting a portion of the muscle, to applying pressure using heavy weight, to using a cold needle to kill muscle fibers, to applying toxins or chemicals (Hardy et al., 2016; Sicherer et al., 2020). The way one introduces injury affects how the muscle is damaged, how much the surrounding cells and tissues are affected and, consequently, the molecular and tissue responses. The variety of injury models corresponds to clinical needs—treatments need to be designed according to the nature and severity of the injury. Even in highly regenerating animal models, the way one inflicts injury influences the extent of regeneration (e.g., in axolotl, see Kragl and Tanaka, 2009; in zebrafish, see Dickover et al., 2013).

**Figure 1.**
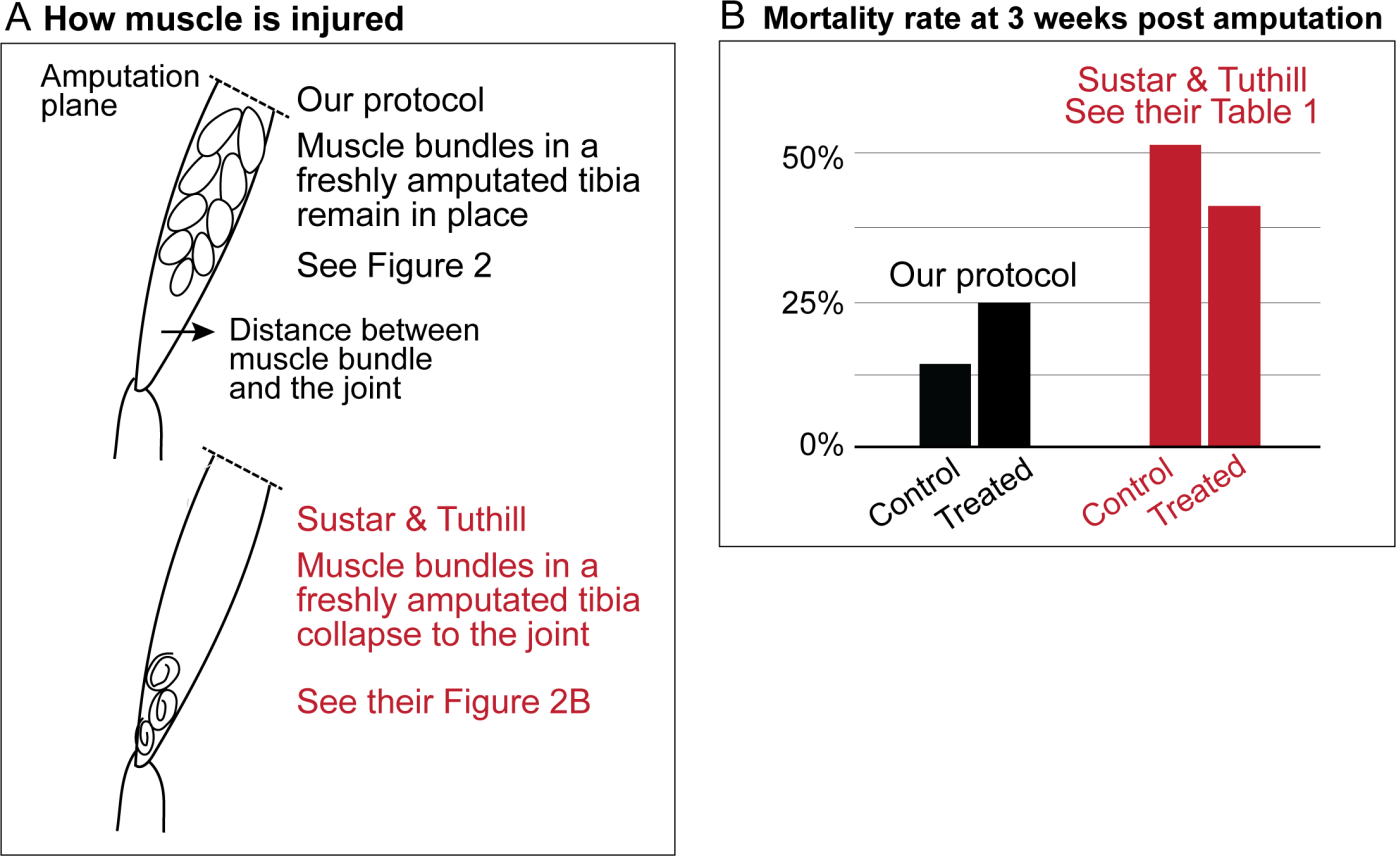
Sustar and Tuthill did not replicate our protocol. (**A**) In our amputation method, muscle bundles in the residual tibia remain in place (see Figure 2). By contrast, in the method used by Sustar and Tuthil, muscle bundles collapsed (see their Figure 2B). (**B**) The lack of stress management in the protocol of Sustar and Tuthill is reflected in the much higher mortality rate in their experiment (as reported in their Table 1).

**Figure 2.**
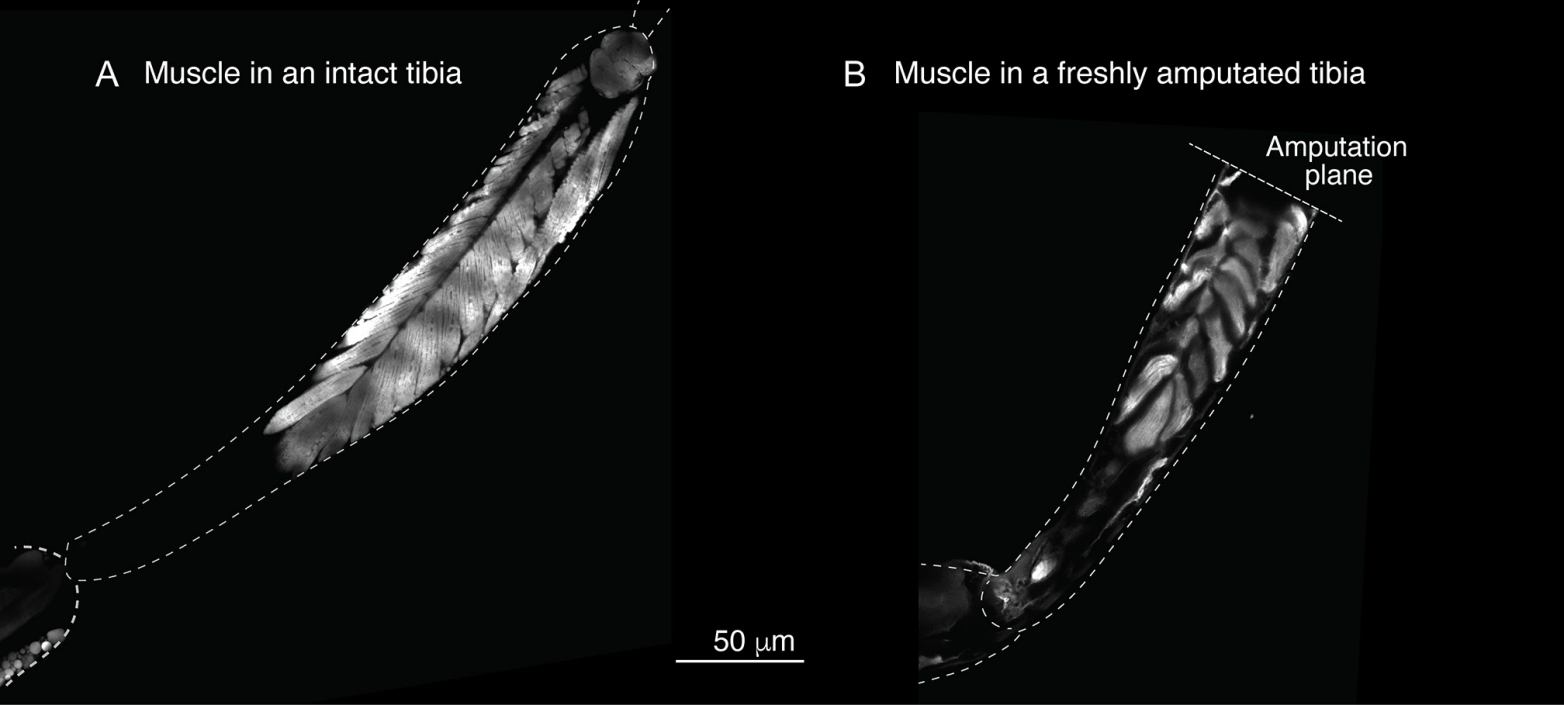
Muscle structure in the limb. To assess the muscle structure in the limb, we analyzed the Mhc-GFP flies, in which promoter of the muscle-specific *myosin heavy chain* (Mhc) gene drives GFP expression. In these images, limbs were dissected and imaged using laser-scanning confocal microscopy.

Sustar and Tuthill used a razor blade to do their amputation (American Safety Razor 72–003). By contrast, as specified in our protocol, we use Vannas surgical scissors (Fine Science Tools 91500–009). The precision of the cutting tips is not the only serious difference. To do a cut with a razor blade, one has to press against the fly leg, thus introducing a different kind of injury. Figures 1a and 2 show how, in our protocol, the muscle in the residual tibias typically looks immediately after amputation. The residual muscle bundles recede a little from the amputation plane and have visibly lost some tension due to the amputation, but they remain largely in place. By contrast, in the work of Sustar and Tuthill (their Figure 2B), the residual muscle bundles in the freshly amputated tibia collapse (see illustrations in Figure 1a).

**Figure 1—figure supplement 1.**
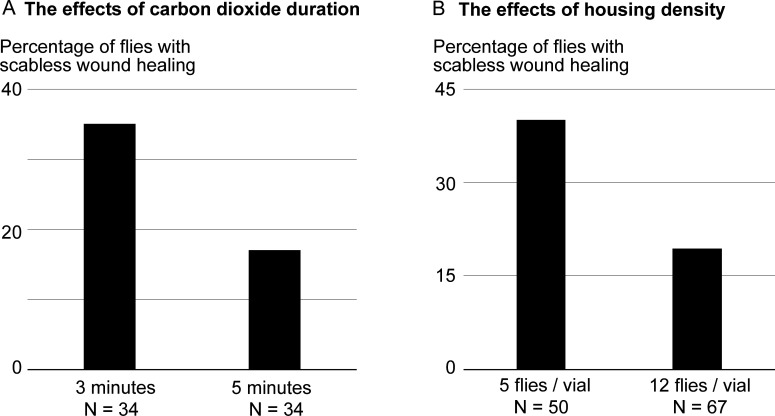
Duration of CO_2_ exposure and housing density can alter the experimental outcomes. In these experiments, adult flies were amputated across the tibia, and placed on food supplemented with leucine, glutamine, and insulin. The effects of the treatment were assessed three days after amputation by the absence of scab formation over the amputation site. (**A**) To perform the amputation, we normally anesthetize the flies using the lowest possible CO_2_ level, and limit anesthesia duration to one to three minutes. Even at thissub-anesthetic CO_2_ level, increasing the CO_2_ exposure time to five minutes is enough to halving the frequency of flies responding to the treatment. (**B**) After amputation, we normally place up to 6 flies per vial. Increasing the housing density reduces the frequency of flies responding to the treatment.

### Age of the flies

Our protocol specifies flies aged between 2 and 7 days old for the amputation, with 3–5 day-olds being the most typical age we use. Sustar and Tuthill used 1–2 day old flies, because “young flies are more likely to regenerate and more likely to survive the three-week recovery period than old flies”. We deliberately avoided using 1-day-old flies because the cuticle is still maturing and extensive tissue remodeling still continues in newly eclosed flies (Kimura and Truman, 1990). As discussed below, the mortality rate in the experiment of Sustar and Tuthill is double the rate from our protocol (Figure 1b).

### Treatment concentration

Sustar and Tuthill used at least three times higher concentrations of insulin and amino acids in their experiment. We take responsibility for this discrepancy: although the correct concentrations were given in the figure legend of Abrams et al., 2021, the concentrations listed in the Materials and methods section of the article were incorrect; we have now published a correction for this article (Abrams et al., 2023).

### Anesthesia protocol

Routine housekeeping work in fly research (e.g., phenotyping, sexing, counting) employs anesthesia. The majority of fly labs anesthetize flies by exposure to carbon dioxide (CO_2_). CO_2_ exposure, however, is known to alter physiological and behavioral processes and is considered stressful, increasing hemolymph acidity and reducing heartbeat (Nicolas and Sillans, 1989; Badre et al., 2005). Stress can impact wound healing and tissue regeneration (Sallin and Jaźwińska, 2016; Hachemi et al., 2018; Huang et al., 2013; Christian et al., 2006). Therefore, we took care to minimize CO_2_ exposure in our protocol. We are not unique in this regard. Protocols in wide-ranging studies from aging to metabolism require minimizing CO_2_ exposure (e.g., Piper and Partridge, 2016; Landis et al., 2020; Colinet and Renault, 2012).

Sustar and Tuthill anesthetized a large group of flies at a time (20 flies), and kept the anesthesia time 5 minutes. By contrast, we anesthetize fewer flies at a time (1–2 flies for the measurements), and keep the anesthesia time much shorter (1–3 minutes). Moreover, we use CO_2_ at a much lower flow rate than that typically used for regular housekeeping. While housekeeping CO_2_ flow rate (~5–10 litres/min) usually puts flies to sleep almost immediately, we reduce CO_2_ flow to the lowest amount possible (2–3 litres/min), just enough to keep the flies from walking—a similar strategy used in other sensitive protocols (e.g., Landis et al., 2020). Increasing the anesthesia time in our protocol is enough to reduce the frequency of flies responding to the treatment (Figure 1—figure supplement 1a).

### Housing density

Housing density is another variable in fly study that can impact various physiological traits, and is a stressor (Nath Das et al., 2022; Joshi and Mueller, 1997). Therefore it is a parameter we optimize in our protocol. Sustar and Tuthill did not specify their housing density, although they processed flies in groups of 20. As specified in our protocol, we house ≤6 flies per vial. Increasing the housing density in our protocol is enough to reduce the frequency of flies responding to the treatment (Figure 1—figure supplement 1b).

These different procedures of stress management result in the very different mortality rates. In the experiment of Sustar and Tuthill, 51% of the control fly amputees died by three weeks post amputation (Figure 1b). By contrast, in our experiment, 14% of the control flies died by three weeks post amputation.

### Regeneration assessment

Sustar and Tuthill argued that our measurements may not be accurate, because “Detecting a subtle phenotype that occurs in only 1% of treated flies would require an exceptional degree of measurement accuracy.” As shown in the measurements to which they refer (Figure 5e in Abrams et al., 2021), 49% of the treated flies showed a change in the residual limb length that is beyond the measurement noise (the 95% confidence intervals of the measurements in control flies). The 1% figure that Sustar and Tuthill cite only describes an earlier experiment (Figure 4) that captures only the most dramatic but rare phenotype, before a more quantitative assay (Figure 5) was performed that captures the fuller extent of the phenotype (49%).

Moreover, the flies that respond to the treatment can be readily identified by eye. Flies that showed limb growth also tend to show a modified wound healing response, apparent within three days after amputation, from the absence of scab formation over the wound. This is a non-trivial phenotype, as scab formation is a genetically controlled process linked to immune signaling (Galko and Krasnow, 2004). Therefore, the response to the treatment occurs in a significant fraction of flies (30–50%), manifests in processes occurring at multiple time scales after injury, and can be screened by eye from the modified wound healing. Facilitated by this ease of scoring responding flies, ongoing work in the lab is dissecting the modified wound environment and characterizing tissue, cellular, and molecular dynamics in the residual limb to understand how nutrient factors modulate the ways with which flies respond to injury.

In some regenerating systems, regeneration proceeds through the formation of a structure called blastema. A blastema is usually composed of dedifferentiated cells that give rise to tissues to replace the lost structures. Sustar and Tuthill argue that there is no blastema in the system. However, blastema characterization was outside the scope of our paper and we did not make any suggestions about a blastema structure.

To assess a blastema formation, Sustar and Tuthill analyzed a white blob phenotype, that occurs in both control and treated flies, and both at the same frequency (3–4%). We do not observe in our protocol a phenotype that occurs in both control and treatment at equal frequency.

## Discussion

In summary, the significant differences in the way Sustar and Tuthill performed the protocol led to serious differences in the nature of the injury and mortality rate, which can explain why they did not see a regenerative response. However, the work of Sustar and Tuthill helped emphasize to us the sensitive parts of the protocol, so we have now published a step-by-step detailed protocol of this work, along with highlights of the critical parameters discussed here (Li et al., 2023).

It is established that adult insects do not regenerate. Sustar and Tuthill argue that adult insects cannot regenerate because they lack the developmental mechanisms to support new growth, due to epigenetic silencing (Harris et al., 2020; Fox et al., 2020). However, the very study they cite (Harris et al., 2020) shows that epigenetic silencing can be modulated. Indeed, multiple studies describe how epigenetic regulations can be modulated, by multiple factors including nutrients (Dai et al., 2020).

Our findings in *Drosophila* add to the increasing body of evidence in the regeneration field that adult animals that do not normally regenerate retain the capacity to activate a regenerative response. As described in the introduction, what is emerging from work across multiple species is that relatively simple interventions can unlock this regenerative capacity, which speaks to us as to the nature of the biological regulations involved. Although the response in flies is far from the dramatic images of regeneration we are used to from the literature in hydras, planarians, and axolotls, the key finding is that a regenerative response can be activated at all. This means we can study the fundamental biological question of what normally prevents these animals from regenerating.

## Methods

### Amputation and treatment

As described in Abrams et al., 2021 and detailed in Li et al., 2023, amputation was performed on adult flies 2–7 days after eclosion. After amputation, flies were reared with the lab food (control) or lab food supplemented with the leucine, insulin and glutamine mix. To make the treared vials, first we made an aqueous master mix of 1.7 mM L-Leucine (Sigma-Aldrich L8912), 1.7 mM L-Glutamine (Sigma-Aldrich G3126), and 0.33 μg/ml insulin (human recombinant, Sigma-Aldrich I0908). Then, we microwaved the fly food in short pulses, to liquify the topmost layer of the food. Microwaving can produce moisture; use a kimwipe to dry the vial wall. Then we pipetted 200 μL of the leucine/insulin/glutamine mix into the liquified layer and mixed with the pipet tip gently. We allowed the food to re-set at 4 °C for at least 20 min, and made sure it was back at room temperature before use to avoid cold-shocking the flies. Flies were kept in treatment for 4–5 days, and then moved to regular food.

### Imaging

For fluorescence imaging, freshly dissected limbs were imaged using the Zeiss LSM 980 laser-scanning confocal microscope, with 63x/1.4 Oil Plan-Apochromat objective. For each limb, multiple images were tiled and stitched using the Zeiss Zen imaging software.

## Data Availability

The raw images of the single fly tracking data are available at CaltechDATA: https://doi.org/10.22002/D1.2157. The raw measurements of the single fly tracking data are available in: https://cdn.elifesciences.org/articles/65092/elife-65092-fig5-data1-v3.xlsx. Matlab code to process the data in the spreadsheet can be downloaded from: https://cdn.elifesciences.org/articles/65092/elife-65092-fig5-code1-v3.zip. The following previously published dataset was used: LiY SarmaA LeeIT CondiotteZ GoentoroL 2021Regeneration data - DrosophilaCaltechDATA10.22002/D1.2157
